# The complete mitochondrial genome sequence and phylogenetic position of *Sinocyclocheilus wumengshanensis* (Cypriniformes: Cyprinidae)

**DOI:** 10.1080/23802359.2017.1407701

**Published:** 2017-11-24

**Authors:** Hongyu Chen, Chunqing Li, Shanyuan Chen, Heng Xiao

**Affiliations:** a School of Life Sciences, Yunnan University, Kunming, China;; b Key Laboratory for Animal Genetic Diversity and Evolution of High Education in Yunnan Province, Yunnan University, Kunming, China

**Keywords:** *Sinocyclocheilus wumengshanensis*, mitogenome sequence, next-generation sequencing

## Abstract

The complete mitochondrial genome (mitogenome) sequence of *Sinocyclocheilus wumengshanensis* from Yunnan Province in China was analysed using next-generation sequencing. The complete mitogenome was 16,585 bp in length and consisted of 13 protein-coding genes, two ribosomal RNA (rRNA) genes, 22 transfer RNA (tRNA) genes, and one D-loop region. Nucleotide composition of the whole mitogenome was 30.5% A, 25.2% T, 27.0% C, and 17.2% G. The gene arrangement and nucleotide composition of the mitogenome of *S. wumengshanensis* were similar to those of other *Sinocyclocheilus* species. Phylogenetic analyses using mitogenomes of 12 species showed that 10 *Sinocyclocheilus* species clustered as one monophyletic clade with strong supports and *S. wumengshanensis* was closely related to *S. grahami*.

*Sinocyclocheilus wumengshanensis* is a species of freshwater fish in the genus *Sinocyclocheilus* (Cypriniformes: Cyprinidae) endemic to China. This species distributes in the karst landform regions within Zhanyi, Xuanwei and Xundian counties of Yunnan province, southwestern China (Li et al. 2003). In this study, we first reported the complete mitochondrial genome (mitogenome) of *S. wumengshanensis*, which could provide useful first-hand data for molecular phylogenetics and population genetics studies on this species and its closely related *Sinocyclocheilus* species.

The specimen of *S. wumengshanensis* was collected in Xuanwei County, Yunnan province, China (26.0°N, 104.0°E). The entire specimen was stored in 95% ethanol and registered in the Zoological Specimen Museum of Yunnan University under the voucher number YNUSM20160817008. Genomic DNA was extracted from muscle tissue by DNeasy Blood & Tissue Kit (QiaGen, Hilden, Germany). Shotgun DNA library was constructed and sequenced with Illumina Miseq platform (Illumina, San Diego, CA). The complete mitogenome sequence was assembled with Trinity v2.3.2 (Haas et al. 2013) and SPAdes (Bankevich et al. 2012). The DOGMA (Wyman et al. 2004) and tRNAscan-SE (Lowe and Eddy 1997) were utilized to annotate protein-coding genes (PCGs), ribosomal RNA (rRNA) genes, and transfer RNA (tRNA) genes, via comparing their similarities to those of *S. grahami* mitogenome sequence (Wu et al. 2010).

The complete mitogenome sequence of *S. wumengshanensis* had been deposited in GenBank database with an accession number MG021442. The complete mitogenome was 16,585 bp in length and consisted of 13 PCGs, two rRNA genes, 22 tRNA genes, and one D-loop or control region. Nucleotide composition of the whole mitogenome was 30.5% A, 25.2% T, 27.0% C, and 17.2% G. The gene arrangement and nucleotide composition of the mitogenome of *S. wumengshanensis* were similar to those of other *Sinocyclocheilus* species (Wu et al. 2010; Li et al. 2017). The locations of 37 genes on H-strand or L-strand and the start codon usage for 13 PCGs were exactly identical to those of *S. jii* mitogenome sequence (Li et al. 2017). For stop codon usage, nine out of 13 PCGs had complete stop codon (TAA or TAG), while four PCGs (COII, ATP8, ND4, and Cyt b) terminated with incomplete stop codon (T-).

To gain an insight into relative phylogenetic position of *S. wumengshanensis*, phylogenetic analyses were conducted based on mitogenome sequences of 10 *Sinocyclocheilus* species and two outgroup species, using Bayesian method by MrBayes3 (Ronquist and Huelsenbeck 2003) and maximum likelihood (ML) method by MEGA6 (Tamura et al. 2013). The two methods generated completely identical tree topologies (Figure 1). The phylogenetic results showed that 10 *Sinocyclocheilus* species clustered as one monophyletic clade with strong supports and that *S. wumengshanensis* was closely related to *S. grahami*.

**Figure 1.**
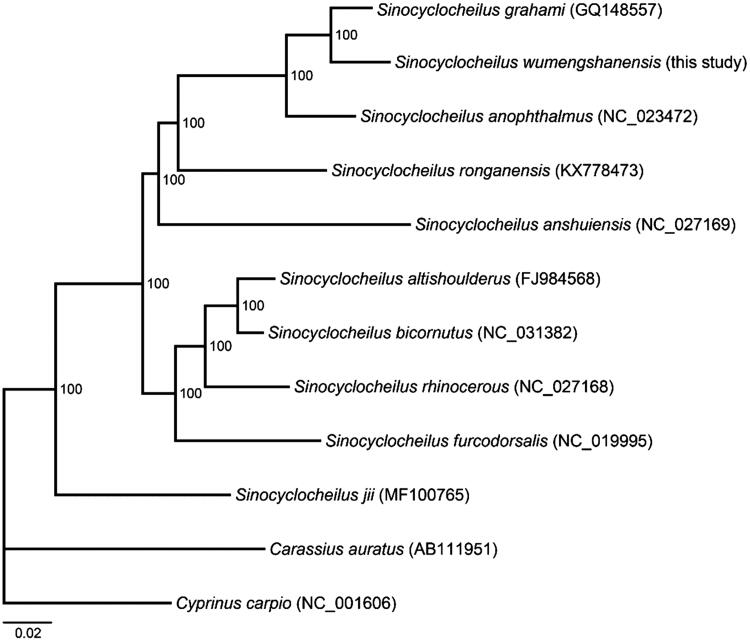
Phylogenetic relationships of 12 Cyprinidae fishes by Bayesian and maximum likelihood (ML) methods based on complete mitochondrial genome sequences. The accession numbers for each species are indicated in brackets. Numbers in the nodes represent support values.
